# Characteristics and in‐hospital outcomes of elderly patients with cancer in a top‐ranked hospital in China, 2016–2020: Real‐world study

**DOI:** 10.1002/cam4.5203

**Published:** 2022-09-26

**Authors:** Lei Huang, Yan Shi, Lei Wang, Lan Rong, Yan Ren, Chenying Xu, Junwei Wu, Mingmin Zhang, Lifeng Zhu, Junjie Zhang, Xiaofeng Xu, Weiguo Hu, Jun Zhang

**Affiliations:** ^1^ Department of Oncology Ruijin Hospital, Shanghai Jiao Tong University School of Medicine Shanghai China; ^2^ Medical Center on Aging of Ruijin Hospital, MCARJH, Shanghai Jiaotong University School of Medicine Shanghai China; ^3^ Department of Gastroenterology Ruijin Hospital, Shanghai Jiaotong University School of Medicine Shanghai People's Republic of China; ^4^ Department of Geriatrics Ruijin Hospital, Shanghai Jiao Tong University School of Medicine Shanghai China; ^5^ Computer Center, Ruijin Hospital Affiliated to Shanghai Jiaotong University School of Medicine Shanghai China; ^6^ BaseBit Technologies, Ltd. Shanghai China; ^7^ Shanghai Chief Technician Studio (Information & Technology) Shanghai China; ^8^ Department of Surgery Ruijin Hospital, Shanghai Jiao Tong University School of Medicine Shanghai China; ^9^ State Key Laboratory of Oncogenes and Related Genes Shanghai Jiao Tong University Shanghai China

**Keywords:** characteristics, cost, duration, geriatric oncology, in‐hospital outcomes, large comprehensive institution‐based study, mortality

## Abstract

**Background:**

Cancer is mostly a disease of aging, and older patients with cancer are generally frailer. This study aimed to describe the characteristics and in‐hospital outcomes and explore factors associated with duration, cost, and mortality during first hospitalization, in older patients with cancer admitted to a top‐ranked hospital in China.

**Methods:**

Data on patients with solid cancer ≥65 years consecutively hospitalized in 2016–2020 were retrieved from the electronic medical records of Ruijin Hospital in Shanghai, China. Baseline characteristics, duration, cost, and mortality during hospitalization were described. Factors associated with duration, cost, and mortality during first hospitalization were explored using multivariable‐adjusted logistic regression.

**Results:**

**20,650** eligible patients with male proportion of 59% and median age of 70 years were analyzed. 45% of the patients underwent resection in our hospital. Upon first admission, 49% of patients had hypertension, 19% diabetes, 22% weight loss, and 28% risks of malnutrition. The median duration and cost of first hospitalization were 9 days and 32,000 RMB, respectively. 118 (0.6%) and 228 (1.1%) deaths occurred during first and any hospitalization, respectively. For first hospitalization, longer duration and higher cost were positively associated with older ages, male gender, emergency admission, certain tumor locations and histology, histories of diabetes, cirrhosis, and anticoagulant intake, higher body mass index, weight loss, reduced food intake, risk of falling, and worse self‐care ability; in‐hospital mortality was positively associated with age ≥85 years, emergency admission, certain cancer types, histories of hypertension and psychotropic intake, reduced food intake, and worse self‐care ability.

**Conclusions:**

This study identified certain baseline patient and tumor characteristics, medical and medication histories, changes of weight and food intake, diet, and self‐care ability which were independently associated with in‐hospital outcomes among older patients with cancer admitted to our hospital and which should be paid special attention to. While the factors might not be easily modifiable, our study can help identify patients at higher risks of inferior in‐hospital outcomes.

## Graphical abstract

This real‐world study identified certain baseline patient and tumor characteristics, medical and medication histories, changes of weight and food intake, diet, and self‐care ability which were independently associated with in‐hospital outcomes among 20,650 older patients (≥65 years) with cancer admitted to a top‐ranked hospital in China during 2016‐2020, and which should be paid special attention to. Our study can help to identify patients at higher risks of inferior in‐hospital outcomes.

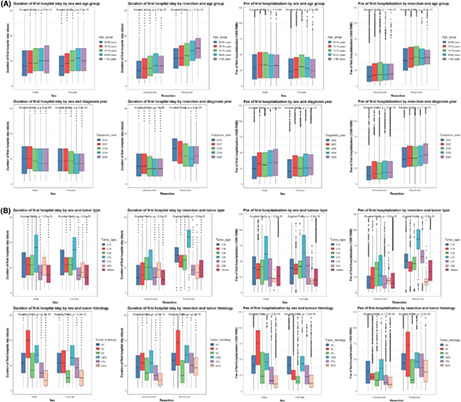

## 1 INTRODUCTION

Cancer is mostly a disease of aging. The number of older adults with cancers (aged ≥65 years) is rapidly increasing and will constitute a growing proportion of the cancer population worldwide. However, older patients remain largely underrepresented in studies which set the standards for cancer management.^1^, ^2^ Notably, older patients with cancer are typically frailer and more vulnerable with more often multiple and complex comorbidities, functional declines, and cognitive impairment, and they continue having inferior outcomes compared to their younger counterparts.^3^, ^4^, ^5^ Managing cancer in the geriatric population can be challenging due to competing aging‐related conditions and mortality risks which can affect treatment decision‐making and impact outcomes.^6^ Older patients with cancer may be under‐treated, and treatment rates significantly decreased with advanced age.^7^, ^8^, ^9^, ^10^, ^11^

Due to the characteristics of older patients with cancer, they may need to stay longer in hospital for management of both cancer and comorbidity, and correspondingly the hospitalization cost may be higher, which may represent a heavier burden for the patients and their families. However, with the increasing coverage of healthcare insurance, the amount and proportion of hospitalization cost paid by the patients themselves may have decreased. The cost issue has been rarely addressed in older cancer patients in China. Peri‐treatment mortality is also significantly higher in older patients.^5^ There have been certain studies reporting in‐hospital outcomes in older patients managed in the US: a retrospective cohort study^12^ using the University Health System Consortium database evaluated the in‐hospital mortality and length of stay in older patients with solid malignancies hospitalized between 1995 and 2003; using the US Nationwide Inpatient Sample (NIS) database, four retrospective studies assessed the inpatient mortality in elderly patients with metastatic brain cancers undergoing resection during 1998–2005,^13^ investigated the in‐hospital mortality in geriatric patients with colorectal cancer undergoing resection between 2005 and 2014,^14^ explored the 30‐day in‐hospital mortality in older patients with pancreatic ductal adenocarcinoma undergoing pancreatoduodenectomy between 2007 and 2010,^15^ and assessed the cost of operative hospitalizations in elderly patients with cancer hospitalized in 2014,^16^ respectively. Few studies addressing the in‐hospital duration, cost, or mortality in older patients in China have been identified.

This study aimed to describe the characteristics and in‐hospital outcomes, including duration, cost, and death, and explore factors associated with duration, cost, and mortality during first hospitalization, in older patients with cancer admitted to a top‐ranked hospital in China. Herein, using the 5‐year data of patients with cancer who were consecutively hospitalized, we comprehensively characterized those ≥65 years and assessed their in‐hospital outcomes; we carefully explored factors associated with the in‐hospital outcomes.

To the best of our knowledge, this real‐world report, which analyzed over 20,000 cases, appeared to be the first and largest institution‐based comprehensive pan‐solid cancer study on characteristics and in‐hospital outcomes, including duration, cost (covered and not covered by insurance), and mortality, of older patients with cancer admitted to a tertiary hospital in China. We further provided findings stratified by gender and resectional surgery and temporal trends of the outcomes, applied the propensity score matching (PSM) method for sensitivity analyses, and performed subgroup analyses for patients admitted through the outpatient route and undergoing planned resectional surgery. Certain baseline patient and tumor characteristics, comorbidities, medical and medication histories, nutrition status, risk assessments, and self‐care ability independently associated with in‐hospital outcomes among older patients with cancer were carefully identified using multivariable‐adjusted analyses and should be paid special attention to; some of the associated factors have not been identified or thoroughly addressed in previous studies. This study can help identify older cancer patients at higher risks of inferior in‐hospital outcomes and requiring more meticulous management to improve in‐hospital outcomes and highlights the importance of careful geriatric assessment and care for older cancer patients.

## 2 METHODS

### 2.1 Patients

The raw record data of each hospitalization of all patients with invasive malignancies who were consecutively hospitalized into Ruijin Hospital, Shanghai Jiao Tong University in Shanghai, China from Jan 1, 2016 to Dec 31, 2020 were retrieved from the Electronic Medical and Nursing Records of Ruijin Hospital with the assistance of the Computer Center. Patient data were prospectively recorded and managed using the Hospital Information System (HIS) in our hospital. Raw records were cleaned and structured into directly analyzable per‐person data with the assistance of BaseBit.AI, using techniques including natural language processing (NLP) with the application of artificial intelligence (AI) methods, including recurrent neural network (RNN).^17^, ^18^ This study was approved by the Ethics Committee of Ruijin Hospital, with informed consent waived. All data were anonymously processed with strict protection of privacy.

In this study, focusing on older patients with solid cancers, we included all malignancies, except those from the hematopoietic and reticuloendothelial systems (International Classification of Diseases for Oncology, Third Edition [ICD‐O‐3] topology code: C42; morphology code: 9590–9989), and only selected patients ≥65 years.^6^ Patients with benign, premalignant, or in situ tumors were excluded.

Information on baseline patient and tumor characteristics, medical and medication histories, nutrition, diet, risk assessments, self‐care ability, duration, cost, and death during hospitalization was collected and referred to that for first admission to or hospitalization in Ruijin Hospital if not otherwise specified. Tumor topology and morphology were (re)coded according to the ICD‐O‐3. The six most common tumor locations in our hospital (stomach, bronchus/lung, colon, pancreas, breast, and rectum) were listed, and the other locations were categorized as others. Among specified histology, the four most frequent broad categories (adenocarcinomas, ductal/lobular cancers, squamous cell cancers, and cystic/mucinous/serous cancers) were listed respectively. Body mass index (BMI; kg/m^2^) was calculated as weight divided by the square of height. Risk of nutrition was assessed using the Nutritional Risk Screening (NRS 2002).^19^ Self‐care ability was quantified using the Barthel index.^20^ Resection referred to that carried out in Ruijin Hospital. Cost was measured using the Chinese Yuan (RMB). Based on the median values, duration and cost (paid by patients) of first hospitalization were dichotomized into ≥ (longer duration) and <10 days (shorter duration), and ≥ (higher cost) and <33,000 RMB (lower cost), respectively. The definitions of the other variables are described in the Appendix S1.

### 2.2 AI

The deep learning method RNN as previously described^21^, ^22^, ^23^ was systematically used to process word segmentation and medical record annotation. The cyclic neural network can make good use of the correlation between adjacent data, so it has been successfully used in a large number of natural language processing (NLP) work for analysis and understanding of information. Therefore, only a small amount of manual annotation was required to complete medical term recognition with the help of RNN's advantages in natural language understanding. Previous studies^21^, ^22^, ^23^ showed that the accuracy of word segmentation using RNN could reach ≥98%, and that the accuracy of medical record annotation could reach ≥97%. After annotation of the medical record by the model, the system generated the original events. The system used the normalization model based on deep learning to normalize the original vectors to form the normalized event vectors. After the abovementioned processes, an original unstructured field was transformed into a group of structured vectors. During the data cleaning process, the system retained the original unstructured fields and well‐structured fields at the same time, which was convenient for flexible use, and which could also allow view of the original unstructured data so as to facilitate regeneration of structured data after the improvement of model accuracy.

### 2.3 Statistics

Categorical variables were shown as count (percentage [%]) and compared between groups using the *χ* ^*2*^ (Mantel–Haenszel *χ* ^*2*^ test^24^, ^25^, ^26^ for 2 × J [J > 2] tables with ordered variables) or Fisher's exact test where appropriate. Continuous variables were presented as mean ± standard deviation; median (interquartile range), and compared between groups using *t* or rank‐sum test where appropriate. We first described the characteristics and in‐hospital outcomes of overall patients and compared them between males and females and between patients undergoing and not undergoing resection. We then used the propensity score matching (PSM) method as previously described^27^, ^28^ for sensitivity analyses, with the matching factors shown in the first column of Table 1 and a caliper of 0.1. Characteristics of patients who died during first or any hospitalization were further described.

**TABLE 1:** Patient and tumor characteristics^a^

Variables	Total	Gender			Resectional surgery		
		Male	Female	*p*	No	Yes	*p*
Baseline patient and tumor characteristics							
*n*	20,650	12,240	8410		11,409	9241	
Year of diagnosis							
2016	3697 (18)	2138 (17)	1559 (19)	0.161	2280 (20)	1417 (15)	0.001
2017	3653 (18)	2180 (18)	1473 (18)		1893 (17)	1760 (19)	
2018	3825 (19)	2279 (19)	1546 (18)		2067 (18)	1758 (19)	
2019	4305 (21)	2555 (21)	1750 (21)		22,389 (20)	2067 (22)	
2020	5170 (25)	3088 (25)	2082 (25)		2931 (26)	2239 (24)	
Gender, male	12,240 (59)	12,240 (100)	0 (0)	—	6689 (59)	5551 (60)	0.036
Age (years)							
As continuous	71 ± 6; 70 (67–75)	71.4 ± 5.5; 70 (67–75)	71.3 ± 5.9; 70 (67–74)	0.001	72 ± 6; 70 (67–75)	71 ± 5; 70 (67–74)	0.019
65–69	9712 (47)	5600 (46)	4112 (49)	0.973	5289 (46)	4423 (48)	<0.001
70–74	5752 (28)	3550 (29)	2202 (26)		3104 (27)	2648 (29)	
75–79	3025 (15)	1908 (16)	1117 (13)		1675 (15)	1350 (15)	
80–84	1496 (7)	859 (7)	637 (8)		899 (8)	597 (6)	
≥85	665 (3)	323 (3)	342 (4)		442 (4)	223 (2)	
Shanghai resident							
Yes	9854 (48)	5441 (44)	4413 (52)	<0.001	5514 (48)	4340 (47)	0.023
No	8976 (43)	5823 (48)	3153 (37)		4864 (43)	4112 (45)	
Unspecified	1820 (9)	976 (8)	844 (10)		1031 (9)	789 (9)	
Admission							
Outpatient	18,216 (88)	10,608 (87)	7608 (90)	<0.001	10,299 (90)	7917 (86)	<0.001
Emergency	2425 (12)	1631 (13)	794 (9)		1103 (10)	1322 (14)	
Others	9 (<1)	1 (<1)	8 (<1)		7 (<1)	2 (<1)	
Tumor location^b^							
C16: Stomach	3238 (16)	2406 (20)	832 (10)	<0.001	1363 (12)	1875 (20)	<0.001
C18: Colon	1973 (10)	1185 (10)	788 (9)		837 (7)	1136 (12)	
C20: Rectum	1366 (7)	902 (7)	464 (6)		951 (8)	415 (4)	
C25: Pancreas	1616 (8)	940 (8)	676 (8)		843 (7)	773 (8)	
C34: Bronchus and lung	2149 (10)	1311 (11)	838 (10)		1324 (12)	825 (9)	
C50: Breast	1382 (7)	21 (<1)	1361 (16)		1077 (9)	305 (3)	
Others	8926 (43)	5475 (45)	3451 (41)		5014 (44)	3912 (42)	
Tumor histology^b^							
814–838: Adenocarcinomas	8675 (42)	5663 (46)	3012 (36)	<0.001	3304 (29)	5371 (58)	<0.001
850–854: Ductal and lobular cancers	2056 (10)	667 (5)	1389 (17)		1166 (10)	890 (10)	
805–808: Squamous cell cancers	1764 (9)	989 (8)	775 (9)		611 (5)	1153 (12)	
844–849: Cystic, mucinous, and serous cancers	1199 (6)	681 (6)	518 (6)		345 (3)	854 (9)	
Others	1339 (6)	889 (7)	450 (5)		868 (8)	471 (5)	
Unspecified	5617 (27)	3351 (27)	2266 (27)		5115 (45)	502 (5)	
Resectional surgery, yes	9241 (45)	5551 (45)	3690 (44)	0.036	0 (0)	9241 (100)	—
Medical and medication histories							
History of hypertension, yes	10,072 (49)	5840 (48)	4232 (50)	<0.001	5430 (48)	4642 (50)	<0.001
History of diabetes, yes	3876 (19)	2243 (18)	1633 (19)	0.048	2150 (19)	1726 (19)	0.760
History of cataract, yes	1786 (9)	861 (7)	925 (11)	<0.001	1037 (9)	749 (8)	0.013
History of coronary heart disease, yes	1222 (6)	727 (6)	495 (6)	0.896	701 (6)	521 (6)	0.133
History of arrhythmia, yes	861 (4)	473 (4)	388 (5)	0.009	483 (4)	378 (4)	0.634
History of stroke, yes	870 (4)	538 (4)	332 (4)	0.124	510 (4.5)	360 (3.9)	0.045
History of cirrhosis, yes	330 (2)	223(2)	107 (1)	0.002	224 (2)	106 (1)	<0.001
History of major abdominal surgery (prior to first hospitalization), yes	5815 (28)	3284 (27)	2531 (30)	<0.001	3915 (34)	1900 (21)	<0.001
History of intake of antihypertensive drug, yes	9393 (45)	5443 (44)	3950 (47)	<0.001	5043 (44)	4350 (47)	<0.001
History of intake of hypoglycemic drugs, yes	3375 (16)	1970 (16)	1405 (17)	0.243	1888 (17)	1487 (16)	0.377
History of intake of anticoagulants, yes	1069 (5)	700 (6)	369 (4)	<0.001	615 (5)	454 (5)	0.131
History of intake of analgesic, yes	582 (3)	363 (3)	219 (3)	0.134	509 (4)	73 (1)	<0.001
History of intake of sedative, yes	421 (2)	217 (1.8)	204 (2.4)	0.001	294 (3)	127 (1)	<0.001
History of intake of psychotropic drug, yes	302 (1)	132 (1)	170 (2)	<0.001	194 (2)	108 (1)	0.002
Changes of weight and food intake, diet, risk assessments, and self‐care ability							
Height (cm)	164 ± 8; 165 (158–170)	169 ± 6; 170 (165–173)	158 ± 5; 158 (155–160)	<0.001	164 ± 8; 165 (158–170)	164 ± 8; 165 (158–170)	0.536
Weight on first admission (kg)	62 ± 11; 62 (55–70)	66 ± 10; 65 (59–72)	58 ± 10; 57 (51–64)	<0.001	62 ± 11; 61 (54–69)	63 ± 11; 63 (56–70)	<0.001
Body mass index on first admission (kg/m^b^)	23 ± 3; 23 (21–25)	23.0 ± 3.3; 22.9 (20.8–25.0)	23.2 ± 3.6; 23.1 (20.8–25.4)	<0.001	22.8 ± 3.5; 22.7 (20.4–25.0)	23.4 ± 3.3; 23.3 (21.2–25.5)	<0.001
Weight loss prior to the first hospitalization^c^, yes	4511 (22)	2974 (24)	1537 (18)	<0.001	2716 (24)	1795 (19)	<0.001
Weight loss value on first admission (kg)^d^	5 ± 3; 4 (2–5)	5 ± 3; 4 (2–5)	4 ± 3; 4 (2–5)	<0.001	5 ± 3; 4 (2–5)	4 ± 3; 4 (2–5)	<0.001
Weight loss percentage on first admission (%)^d^	5 ± 3; 4 (2–5)	5 ± 3; 4 (2–5)	4 ± 3; 4 (2–5)	0.043	5 ± 3; 4 (2–5)	4 ± 3; 4 (2–5)	<0.001
Weight loss duration on first admission (months)^d^	2 ± 1; 2 (1–3)	2 ± 1; 2 (1–3)	2 ± 1; 2 (1–3)	0.516	2 ± 1; 2 (1–3)	2 ± 1; 2 (1–3)	0.245
Percentage of reduced food intake within 1 week of first admission (%)^e^							
0	18,070 (88)	10,629 (87)	7441 (89)	0.001	9758 (86)	8312 (90)	<0.001
1–50	1725 (8)	1091 (9)	634 (8)		1060 (9)	665 (7)	
51–75	586 (3)	353 (3)	233 (3)		395 (3)	191 (2)	
76–100	159 (1)	105 (1)	54 (1)		100 (1)	59 (1)	
Basic diet on first admission							
Common diet	14,137 (68)	8379 (68)	5758 (68)	0.003	7268 (64)	6869 (74)	<0.001
Soft or semi‐fluid diet	2743 (13)	1791 (15)	952 (11)		1690 (15)	1053 (11)	
Others	3770 (18)	2070 (17)	1700 (20)		2451 (21)	1319 (14)	
Low‐salt diet on first admission, yes	2497 (12)	1404 (11)	1093 (13)	0.001	1593 (14)	904 (10)	<0.001
Diabetes diet on first admission, yes	2257 (11)	1291 (10.6)	966 (11.5)	0.036	1373 (12)	884 (10)	<0.001
Risk of malnutrition on first admission^f^, yes	5668 (28)	3404 (28)	2264 (27)	0.166	3686 (33)	1982 (21)	<0.001
Risk of falling on first admission^g^, yes	1733 (8)	943 (8)	790 (9)	<0.001	996 (9)	737 (8)	0.039
Barthel index for self‐care ability on first admission	96 ± 14; 100 (100–100)	96 ± 14; 100 (100–100)	95 ± 15; 100 (100–100)	0.345	94 ± 17; 100 (100–100)	98 ± 10; 100 (100–100)	<0.001

^a^ Categorical variables are shown as count (percentage [%]), and are compared between groups using *χ* ^*b*^ or Fisher's exact test where appropriate. Continuous variables are shown as mean ± standard deviation; median (interquartile range), and are compared between groups using the Wilcoxon rank‐sum test (Mann–Whitney *U* test). Admission and resection referred to those to/in Ruijin Hospital, Shanghai Jiao Tong University School of Medicine in Shanghai, China.

^b^ Coded according to the International Classification of Diseases for Oncology, Third Edition (ICD‐O‐3).

^c^ Unknown weight loss prior to the first hospitalization: total, 109 (1%); male, 62 (1%); female, 47 (1%); unresected, 95 (1%); resected, 14 (<1%).

^d^ Calculated for cases with weight loss on first admission only.

^e^ Unknown percentage of reduced food intake within 1 week of first admission: total, 110 (1%); male, 62 (1%); female, 48 (1%); unresected, 96 (1%); resected, 14 (<1%).

^f^ Unknown risk of malnutrition on first admission: total, 109 (1%); male, 62 (1%); female, 47 (1%); unresected, 95 (1%); resected, 14 (<1%).

^g^ Unknown risk of falling on admission for surgery: total, 109 (1%); male, 62 (1%); female, 47 (1%); unresected, 95 (1%); resected, 14 (<1%).

The overall and stratified correlations between duration and cost of first hospitalization were illustrated, with the corresponding correlation coefficients computed. We then plotted the duration and cost according to age group, year of diagnosis, cancer type, and histology, with stratification by gender and resection. Factors associated with duration (≥ vs. <10 days), cost (≥ vs. <33,000 RMB), and death (yes vs. no) during first hospitalization were further explored using multivariable logistic regression with adjustment for multiple patient and tumor features, and with the odds ratios (ORs) and corresponding 95% confidence intervals (CIs) calculated. All available variables (Table 1) including baseline patient and tumor characteristics, medical and medication histories, changes of body mass index and food intake, diet, risk assessments, and self‐care ability were included in the multivariable model.

Data were analyzed using R 4.1.1 (https://www.r‐project.org/) in the XDP forum by BaseBit.AI, and a 2‐sided *p* value <0.05 indicated statistical significance.

## 3 RESULTS

### 3.1 Overall and stratified characteristics

64,030 person‐time data (in the raw records, recurring hospitalizations were accounted for individually) of hospitalized patients who were ≥65 years with solid cancers and with diagnosis from Jan 1, 2016 through Dec 31, 2020 were exported from the Computer Center of our hospital and were then cleaned into records for 21,286 patients. After excluding 636 patients with benign, premalignant, or in situ tumors, 20,650 patients with invasive cancers were analyzed.

Baseline patient and tumor characteristics are shown in Table 1. Males accounted for 59% of the total patients, and the median age was 70 years. Case number increased from 3697 in 2016 to 5170 in 2020. 48% of the patients resided in Shanghai (Shanghai residents regardless of registered permanent residence in Shanghai and medical insurance status), and 88% were admitted through the outpatient route (planned admissions through outpatient service as opposed to emergency admission), with the others mostly through the emergency pathway. The most frequently diagnosed cancer in our hospital was stomach cancer (16%), followed by bronchus/lung (10%) and colon cancers (10%). The most common histology was adenocarcinoma (42%). 45% of the patients underwent resection in our hospital.

Compared to male patients, females more often resided in Shanghai (52% vs. 44%) and were less frequently admitted through the emergency pathway (9% vs. 13%). Female patients had less often stomach cancers (10% vs. 20%) and adenocarcinomas (36% vs. 46%) but more often breast cancers (16% vs. <1%) and ductal/lobular cancers (17% vs. 5%).

Relative to patients not undergoing resection in our hospital, those resected in our hospital were less often ≥80 years (8% vs. 12%) and had more frequently emergency admission (14% vs. 10%); they had greater proportions of stomach (20% vs. 12%) and colon cancers (12% vs. 7%), but smaller proportions of rectum (4% vs. 8%) and breast cancers (3% vs. 9%).

The medical and medication histories, nutrition, risk assessments, and self‐care ability, both overall and stratified by gender and resection in our hospital, and characteristics and in‐hospital outcomes of resected patients on admission for surgery are shown in Table 1 and Table S1 and described in Appendix S1.

### 3.2 In‐hospital outcomes

The median durations of first and total hospital stay of all the patients during the analyzed period were 9 and 13 days, respectively, and the median costs of first and all hospitalization were 32,000 and 44,000 RMB, respectively (Table 2). The median costs not covered by insurance for first and all hospitalization were 11,000 and 17,000 RMB, respectively. One hundred and eighteen (0.6%) and 228 (1.1%) of patients died during first and any hospitalization, and their characteristics are detailed in Table S2.

**TABLE 2:** Hospitalization, costs, and in‐hospital deaths^a^

Variable	Total	Gender			Resectional surgery		
		Male	Female	*p*	No	Yes	*p*
*n*	20,650	12,240	8410		11,409	9241	
Number of hospitalizations	1 (1–2)	1 (1–3)	1 (1–2)	<0.001	1 (1–3)	1 (1–2)	<0.001
Days of first hospital stay							
As continuous	9 (5–15)	9 (5–15)	9 (5–14)	<0.001	7 (3–12)	12 (8–17)	<0.001
≥10	10,012 (48)	6077 (50)	3935 (47)	<0.001	4051 (36)	5961 (65)	<0.001
Days of total hospital stay	13 (7–22)	14 (8–23)	12 (7–21)	<0.001	11 (6–21)	15 (9–23)	<0.001
Costs of first hospitalization (×1000 RMB)							
As continuous	32 (12–52)	33 (12–55)	26 (12–47)	<0.001	17 (8–37)	44 (29–64)	<0.001
≥33	9564 (46)	6062 (50)	3502 (42)	<0.001	3227 (28)	6337 (69)	<0.001
Total costs of any hospitalization (×1000 RMB)	44 (22–74)	47 (26–78)	38 (19–27)	<0.001	32 (15–65)	54 (35–81)	<0.001
Costs not covered by insurance for first hospitalization (×1000 RMB)	11 (2–33)	12 (3–35)	11 (1–29)	<0.001	7 (1–19)	22 (7–44)	<0.001
Costs not covered by insurance for first hospitalization/costs of first hospitalization (%)	56 (9–100)	66 (12–100)	48 (6–100)	<0.001	83 (3–100)	52 (18–100)	<0.001
Total costs not covered by insurance for any hospitalization (×1000 RMB)	17 (4–42)	19 (5–44)	14 (2–38)	<0.001	11 (1–32)	27 (10–52)	<0.001
Total costs not covered by insurance for any hospitalization/total costs of any hospitalization (%)	47 (10–100)	51 (12–100)	41 (7–100)	<0.001	45 (4–100)	48 (17–100)	<0.001
Death during first hospitalization, yes	118 (1)	65 (1)	53 (1)	0.350	102 (1)	16 (<1)	<0.001
Death during any hospitalization, yes	228 (1)	131 (1)	97 (1)	0.588	192 (2)	36 (<1)	<0.001

^a^ Categorical variables are shown as count (percentage [%]), and were compared between groups using *χ* ^*2*^ or Fisher's exact test where appropriate. Continuous variables are shown as median (interquartile range) considering the data distribution and were compared between groups using the Wilcoxon rank‐sum test (Mann–Whitney *U* test). Admission and resection referred to those to/in Ruijin Hospital, Shanghai Jiao Tong University School of Medicine in Shanghai, China.

Compared to male patients, females less often had duration of first hospital stay ≥10 days (47% vs. 50%) and paid less for first hospitalization (median, 26,000 vs. 33,000 RMB), with smaller proportions of ≥33,000 RMB (42% vs. 50%). Accordingly, females had shorter duration of total hospital stay (12 vs. 14 days) and also paid less in total for any hospitalization (median, 38,000 vs. 47,000 RMB). The median costs not covered by insurance for first (11,000 vs. 12,000 RMB) and any hospitalization (14,000 vs. 19,000 RMB) were slightly lower for women than for men.

Relative to patients not undergoing resection in our hospital, those resected in our hospital had longer duration of first (median, 12 vs. 7 days) and total hospital stay (median, 15 vs. 11 days), with a markedly higher proportion of first hospital stay for ≥10 days (65% vs. 36%); they also paid more for first (median, 44,000 vs. 17,000 RMB; proportions of ≥33,000 RMB, 69% vs. 28%) and any hospitalization (54,000 vs. 32,000 RMB). The median costs not covered by insurance for first (22,000 vs. 7000 RMB) and any hospitalization (27,000 vs. 11,000 RMB) were also higher for patients undergoing resection. Resected patients less often died in hospital during first (0.2% vs. 0.9%) and any hospitalization (0.4% vs. 1.7%).

From 2016 to 2020, the median duration of first hospital stay decreased from 14 to 11 days for patients who underwent resectional surgery and from 7 to 6 days for those who did not; the mortality rate for first hospitalization decreased from 0.4% to 0.2% for patients who underwent resectional surgery and from 1.2% to 0.6% for those who did not.

PSM analyses were further performed, and the characteristics were comparable between males and females and between patients who underwent resectional surgery and those who did not after matching (Table S3). After matching, the inter‐gender differences in number of hospitalizations, proportion of higher cost of first hospitalization, cost not covered by insurance for first hospitalization, and total cost not covered by insurance for any hospitalization became insignificant compared to the findings before matching (Table S4). The results on comparisons of in‐hospital outcomes according to resection status remained similar with the findings before matching (Table S4).

### 3.3 Stratification analyses of duration and cost of first hospitalization

**FIGURE 1:**
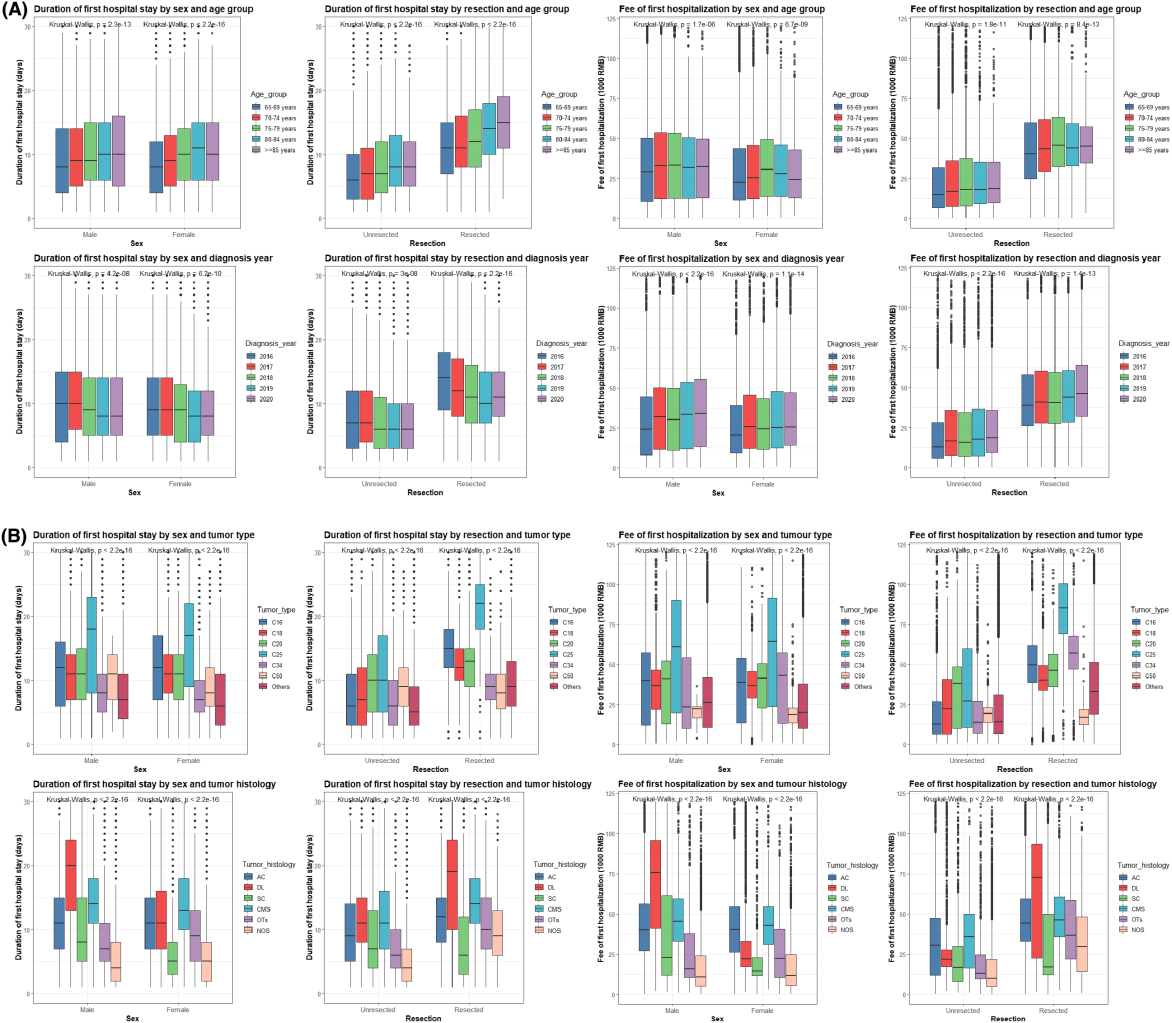
Associations of duration and cost of first hospitalization with age group, year of diagnosis, cancer type, and histology in patients with cancer ≥65 years, stratified by gender and resection. Tumor topology and morphology were (re)coded according to the International Classification of Diseases for Oncology, Third Edition (ICD‐O‐3). C16, stomach; C18, colon; C20, rectum; C25, pancreas; C34, bronchus/lung; C50, breast; AC, adenocarcinoma; DL, ductal/lobular cancer; SC, squamous cell cancer; CMS, cystic/mucinous/serous cancer; OTs, others; NOS, not otherwise specified; RMB, Chinese yuan.

Duration and cost of first hospitalization were closely correlated with each other (overall *r* = 0.754). The correlation was stronger in male and unresected patients (both *r* = 0.762) and was strongest in patients ≥85 years (*r* = 0.770), in patients diagnosed in 2020 (*r* = 0.785), for pancreas cancers (*r* = 0.826), and for ductal/lobular cancers (*r* = 0.846). We further plotted the duration and cost of first hospitalization against age group, year of diagnosis, cancer type, and histology, with stratification by gender and resection (Figure 1), and the findings are described in Appendix S1. Regardless of gender and resection in our hospital, duration of first hospital stay decreased, while cost increased in more recent years.

### 3.4 Multivariable analyses of factors associated with duration, cost, and death of first hospitalization

The proportion of longer first hospital stay decreased in more recent years (e.g., OR_2020 vs. 2016_ = 0.62) and increased with older ages (e.g., OR_≥85 vs. 65–69 year_ = 1.60; Table 3). Female patients less frequently had longer stay (OR = 0.91). Patients who did not reside in Shanghai (OR = 1.17) and those who were admitted through the emergency pathway (OR = 1.69) more likely had longer first hospital stay. Compared to stomach cancer, rectum (OR = 1.23) and pancreas cancers (OR = 1.75) were associated with more often longer first hospital stay, while bronchus/lung (OR = 0.40) and breast cancers (OR = 0.50) with less frequently longer stay. Relative to patients with adenocarcinomas, those with ductal/lobular (OR = 2.24) and cystic/mucinous/serous cancers (OR = 1.81) had more often longer first hospital stay, opposite to those with squamous cell cancers (OR = 0.53). Patients undergoing resection in our hospital more likely had longer first hospital stay (OR = 2.42), opposite to those with histories of major abdominal surgeries (OR = 0.70). Histories of diabetes (OR = 1.27), cataract (OR = 1.23), and cirrhosis (OR = 3.87) were associated with more often longer first hospital stay. Patients with histories of anticoagulant intake had more frequently longer first hospital stay (OR = 1.30), opposite to those with histories of sedative intake (OR = 0.55). Patients with weight loss (OR = 1.36) and reduced food intake (e.g., OR_76‐100% vs. 0%_ = 1.69) had more often longer first hospital stay. Low‐salt (OR = 0.77) and diabetes diets (OR = 0.72) and higher Barthel Index for better self‐care ability (OR = 0.99) were associated with less often longer first hospital stay.

**TABLE 3:** Factors associated with duration of first hospital stay, costs of first hospitalization, and death during first hospitalizations^a^

Variable	Days of first hospital stay (≥ vs. <10)			Costs of first hospitalization (≥ vs. <33,000 RMB)			Death during first hospitalization (yes vs. no)		
	OR (95% CI)	*p*	*p*_*trend*_	OR (95% CI)	*p*	*p*_*trend*_	OR (95% CI)	*p*	*p*_*trend*_
Days of first hospital stay (ref.: <10)									
≥10	—	—		12.55 (11.51–13.67)	**<0.001**		—	—	
Year of diagnosis (ref.: 2016)			**<0.001**			**<0.001**			0.352
2017	0.84 (0.75–0.93)	**0.001**		1.44 (1.26–1.64)	**<0.001**		0.74 (0.41–1.33)	0.315	
2018	0.64 (0.57–0.71)	**<0.001**		1.57 (1.38–1.79)	**<0.001**		0.72 (0.40–1.31)	0.279	
2019	0.55 (0.49–0.61)	**<0.001**		2.24 (1.97–2.54)	**<0.001**		0.53 (0.29–0.99)	**0.048**	
2020	0.62 (0.56–0.69)	**<0.001**		2.76 (2.44–3.13)	**<0.001**		0.65 (0.36–1.17)	0.152	
Gender (ref.: Male)									
Female	0.91 (0.84–0.97)	**0.005**		1.00 (0.92–1.08)	0.931		0.99 (0.66–1.48)	0.945	
Age (years; ref.: 65–69)			**<0.001**			**<0.001**			**0.035**
70–74	1.17 (1.08–1.27)	**<0.001**		1.24 (1.12–1.36)	**<0.001**		0.85 (0.47–1.54)	0.594	
75–79	1.35 (1.22–1.49)	**<0.001**		1.23 (1.09–1.39)	**0.001**		1.74 (0.99–3.06)	0.054	
80–84	1.58 (1.38–1.81)	**<0.001**		1.09 (0.92–1.28)	0.317		1.55 (0.81–2.97)	0.190	
≥85	1.60 (1.32–1.94)	**<0.001**		1.38 (1.09–1.75)	**0.008**		2.28 (1.13–4.62)	**0.022**	
Shanghai resident (ref.: Yes)			**<0.001**			**<0.001**			**<0.001**
No	1.17 (1.09–1.25)	**<0.001**		1.36 (1.25–1.48)	**<0.001**		0.18 (0.09–0.35)	**<0.001**	
Unspecified	1.01 (0.90–1.13)	0.878		1.16 (1.01–1.34)	**0.038**		1.32 (0.80–2.18)	0.281	
Admission (ref.: Outpatient)			**<0.001**			0.269			**0.001**
Emergency	1.69 (1.52–1.88)	**<0.001**		1.03 (0.92–1.17)	0.591		2.46 (1.56–3.88)	**<0.001**	
Others	1.09 (0.20–5.86)	0.918		0.15 (0.01–1.72)	0.127		—	—	
Tumor location^b^ (ref.: C16: Stomach)			**<0.001**			**<0.001**			**0.001**
C18: Colon	1.03 (0.90–1.18)	0.681		1.51 (1.28–1.78)	**<0.001**		0.70 (0.30–1.59)	0.390	
C20: Rectum	1.23 (1.06–1.43)	**0.006**		2.99 (2.48–3.59)	**<0.001**		0.10 (0.01–0.79)	**0.029**	
C25: Pancreas	1.75 (1.47–2.08)	**<0.001**		3.09 (2.50–3.82)	**<0.001**		2.38 (1.08–5.25)	**0.031**	
C34: Bronchus and lung	0.40 (0.35–0.46)	**<0.001**		3.28 (2.80–3.86)	**<0.001**		1.11 (0.51–2.39)	0.799	
C50: Breast	0.50 (0.41–0.61)	**<0.001**		0.05 (0.03–0.07)	**<0.001**		0.12 (0.01–1.05)	0.055	
Others	0.49 (0.44–0.54)	**<0.001**		1.23 (1.08–1.40)	**0.002**		0.81 (0.42–1.58)	0.539	
Tumor histology^b^ (ref.: 814–838: Adenocarcinomas)			**<0.001**			**<0.001**			0.241
850–854: Ductal and lobular cancers	2.24 (1.90–2.64)	**<0.001**		0.93 (0.75–1.15)	0.500		0.84 (0.35–2.04)	0.705	
805–808: Squamous cell cancers	0.53 (0.47–0.60)	**<0.001**		0.30 (0.26–0.35)	**<0.001**		1.56 (0.68–3.57)	0.295	
844–849: Cystic, mucinous, and serous cancers	1.81 (1.54–2.12)	**<0.001**		1.16 (0.97–1.38)	0.101		0.50 (0.12–2.11)	0.342	
Others	0.54 (0.47–0.62)	**<0.001**		0.45 (0.38–0.52)	**<0.001**		0.67 (0.33–1.38)	0.277	
Unspecified	0.28 (0.25–0.30)	**<0.001**		0.41 (0.37–0.46)	**<0.001**		0.64 (0.39–1.06)	0.082	
Resectional surgery (ref.: No)									
Yes	2.42 (2.24–2.60)	**<0.001**		3.46 (3.17–3.78)	**<0.001**		0.24 (0.13–0.43)	**<0.001**	
History of hypertension (ref.: No)									
Yes	1.09 (0.94–1.27)	0.268		1.11 (0.92–1.34)	0.266		3.34 (1.62–6.91)	**0.001**	
History of diabetes (ref.: No)									
Yes	1.27 (1.04–1.57)	**0.023**		1.08 (0.84–1.39)	0.551		1.31 (0.54–3.16)	0.551	
History of cataract (ref.: No)									
Yes	1.23 (1.10–1.39)	**0.001**		1.03 (0.90–1.19)	0.651		1.23 (0.71–2.12)	0.470	
History of coronary heart disease (ref.: No)									
Yes	1.13 (0.98–1.31)	0.090		1.05 (0.88–1.24)	0.605		0.91 (0.42–1.98)	0.815	
History of arrhythmia (ref.: No)									
Yes	1.17 (1.00–1.38)	0.057		0.90 (0.73–1.09)	0.276		1.15 (0.57–2.35)	0.692	
History of stroke (ref.: No)									
Yes	1.17 (0.99–1.39)	0.065		0.87 (0.71–1.06)	0.161		0.86 (0.42–1.77)	0.680	
History of cirrhosis (ref.: No)									
Yes	3.87 (2.99–5.00)	**<0.001**		1.03 (0.77–1.38)	0.844		0.20 (0.02–1.99)	0.172	
History of major abdominal surgery (ref.: No)									
Yes	0.70 (0.64–0.76)	**<0.001**		0.83 (0.75–0.92)	**<0.001**		1.24 (0.77–1.99)	0.379	
History of intake of antihypertensive drug (ref.: No)									
Yes	1.01 (0.87–1.18)	0.877		0.96 (0.80–1.16)	0.663		0.48 (0.24–0.97)	**0.042**	
History of intake of hypoglycemic drug (ref.: No)									
Yes	1.03 (0.84–1.27)	0.771		1.02 (0.79–1.31)	0.904		0.91 (0.36–2.28)	0.835	
History of intake of anticoagulants (ref.: No)									
Yes	1.30 (1.11–1.53)	**0.001**		1.04 (0.86–1.25)	0.699		0.39 (0.14–1.08)	0.068	
History of intake of analgesic (ref.: No)									
Yes	0.96 (0.78–1.19)	0.716		0.43 (0.33–0.56)	**<0.001**		1.56 (0.76–3.21)	0.224	
History of intake of sedative (ref.: No)									
Yes	0.55 (0.43–0.70)	**<0.001**		1.04 (0.78–1.40)	0.788		—	—	
History of intake of psychotropic drug (ref.: No)									
Yes	1.28 (0.97–1.68)	0.081		0.67 (0.47–0.93)	**0.018**		4.46 (2.11–9.42)	**<0.001**	
Body mass index on first admission (as continuous)	1.00 (0.99–1.01)	0.463		1.02 (1.01–1.03)	**0.008**		0.96 (0.90–1.02)	0.156	
Weight loss on first admission (ref.: No)									
Yes	1.36 (1.24–1.49)	**<0.001**		0.95 (0.85–1.05)	0.303		0.80 (0.48–1.31)	0.366	
Percentage of reduced food intake within 1 week of first admission (%; ref.: 0)			**<0.001**			0.239			**0.002**
1–50	1.34 (1.18–1.53)	**<0.001**		0.91 (0.78–1.05)	0.207		2.02 (1.12–3.62)	**0.019**	
51–75	1.58 (1.27–1.96)	**<0.001**		1.00 (0.78–1.27)	0.972		3.70 (1.88–7.28)	**<0.001**	
76–100	1.69 (1.12–2.54)	**0.012**		1.40 (0.90–2.19)	0.139		1.92 (0.68–5.46)	0.220	
Basic diet on first admission (ref.: Common diet)			0.262			**<0.001**			0.064
Soft or semi‐fluid diet	0.93 (0.83–1.03)	0.141		1.31 (1.16–1.48)	**<0.001**		0.76 (0.42–1.38)	0.369	
Others	1.03 (0.91–1.17)	0.672		1.10 (0.95–1.28)	0.205		1.63 (0.93–2.85)	0.089	
Low‐salt diet on first admission (ref.: No)									
Yes	0.77 (0.67–0.88)	**<0.001**		0.62 (0.52–0.73)	**<0.001**		0.57 (0.28–1.17)	0.124	
Diabetes diet on first admission (ref.: No)									
Yes	0.72 (0.61–0.84)	**<0.001**		0.88 (0.72–1.07)	0.195		0.90 (0.40–2.03)	0.801	
Risk of malnutrition on first admission (ref.: No)									
Yes	0.99 (0.90–1.09)	0.818		0.83 (0.74–0.93)	**0.002**		0.98 (0.57–1.70)	0.948	
Risk of falling on first admission (ref.: No)									
Yes	1.03 (0.91–1.16)	0.673		1.31 (1.13–1.52)	**<0.001**		1.63 (0.98–2.71)	0.058	
Barthel index for self‐care ability on first admission (as continuous)	0.99 (0.99–0.99)	**<0.001**		1.00 (1.00–1.01)	0.136		0.98 (0.97–0.98)	**<0.001**	

Abbreviations: −, not estimable; CI, confidence interval; OR, odds ratio; ref., reference.

^a^ The ORs and the corresponding 95% CI for the associations of ≥ versus <10 days of first hospital stay, ≥ versus <33,000 RMB as costs for first hospitalization, and death versus survival during first hospitalization with the factors listed in the first column of the table were estimated using the multivariable‐adjusted logistic regression model with mutual adjustment for the variables listed in the first column. Admission and resection referred to those to/in Ruijin Hospital, Shanghai Jiao Tong University School of Medicine in Shanghai, China. *p* values which are <0.05 are shown in bold and indicate statistical significance.

^b^ Coded according to the International Classification of Diseases for Oncology, Third Edition (ICD‐O‐3).

Patients with duration of first hospital stay ≥10 days had significantly more often cost of first hospitalization ≥33,000 RMB (OR = 12.55). Patients diagnosed in more recent years (e.g., OR_2020 vs. 2016_ = 2.76) and older patients (e.g., OR_≥85 vs. 65–69 year_ = 1.38) had more often higher costs, and costs were more frequently higher for patients residing outside of Shanghai (OR = 1.36). Compared to stomach cancer, colon (OR = 1.51), rectum (OR = 2.99), pancreas (OR = 3.09), and bronchus/lung cancers (OR = 3.28) were associated with more often higher costs, opposite to breast cancer (OR = 0.05). Relative to patients with adenocarcinomas, those with squamous cell cancers (OR = 0.30) had less often higher costs. Resected patients more likely had higher costs (OR = 3.46), opposite to those with histories of major abdominal surgery (OR = 0.83). Histories of analgesic (OR = 0.43) and psychotropic drug intake (OR = 0.67) were significantly associated with less often higher costs. Patients with higher BMI had more likely higher costs (OR = 1.02). Patients having soft or semi‐fluid diet had more often higher costs than those having common diet (OR = 1.31). Low‐salt diet (OR = 0.62) was associated with less frequently higher costs, while risk of falling with more often higher costs (OR = 1.31).

Patients 85 years of age or older died in the hospital more often than those 65–69 years old (OR = 2.28). Patients residing outside of Shanghai less often died during first hospitalization (OR = 0.18), while those admitted through emergency pathways died more frequently in the hospital (OR = 2.46). Compared to patients with stomach cancer, those with rectum cancer less frequently died in the hospital (OR = 0.10), while those with pancreas cancer died more often (OR = 2.38). Resected patients less often died during first hospitalization (OR = 0.24). Patients with a history of hypertension died more often in the hospital (OR = 3.34), while those having intake of antihypertensive drug died less frequently (OR = 0.48). Patients with a history of psychotropic drug intake markedly more frequently died in the hospital (OR = 4.46). Patients with reduced food intake within 1 week (determined by medical history only) of first admission more frequently died during first hospitalization (e.g., OR_51‐75% vs. 0%_ = 3.70), while those with a higher Barthel Index for better self‐care ability died less often (OR = 0.98).

Subgroup analyses for patients admitted through the outpatient route and undergoing planned resectional surgery are shown in Table 4. Since there were only 10 (0.1%) patients among the subgroup who died during the first hospitalization and the corresponding multivariable model did not converge, results for death during first hospitalization were not shown. Compared to the findings in the overall patients, results for duration and cost of first hospitalization were mostly similar in pattern, with some changes in association strength and significance and some exceptions. For example, both colon (OR = 0.70) and rectum (OR = 0.44) cancers became significantly associated with less often longer first hospital stay, relative to stomach cancer, and the associations of length of hospital stay with histories of diabetes, major abdominal surgery, and intake of sedative became insignificant. Female gender became significantly associated with lower cost of first hospitalization (OR = 0.73), and the associations of cost with both colon and rectum cancers, histories of major abdominal history and analgesic and psychotropic drug intakes, and low‐salt diet became insignificant. A higher BMI became significantly associated with less frequently higher cost (OR = 0.97).

**TABLE 4:** Factors associated with duration of first hospital stay, costs of first hospitalization, and death during first hospitalizations in patients admitted through the outpatient route and undergoing planned resectional surgery^a^

Variable	Days of first hospital stay (≥ vs. <10)			Costs of first hospitalization (≥ vs. <33,000 RMB)		
	OR (95% CI)	*p*	*p*_*trend*_	OR (95% CI)	*p*	*p*_*trend*_
Days of first hospital stay (ref.: <10)						
≥10	—	—		12.91 (11.15–14.95)	**<0.001**	
Year of diagnosis (ref.: 2016)			**<0.001**			**<0.001**
2017	0.68 (0.56–0.82)	**<0.001**		1.22 (0.99–1.51)	0.069	
2018	0.48 (0.40–0.58)	**<0.001**		1.40 (1.13–1.74)	**0.002**	
2019	0.40 (0.34–0.49)	**<0.001**		2.59 (2.08–3.23)	**<0.001**	
2020	0.52 (0.43–0.62)	**<0.001**		3.42 (2.74–4.28)	**<0.001**	
Gender (ref.: Male)						
Female	0.81 (0.73–0.91)	**<0.001**		0.73 (0.64–0.84)	**<0.001**	
Age (years; ref.: 65–69)			**<0.001**			0.193
70–74	1.17 (1.03–1.33)	**0.020**		1.20 (1.02–1.40)	**0.028**	
75–79	1.26 (1.06–1.49)	**0.008**		1.20 (0.98–1.47)	0.085	
80–84	1.96 (1.52–2.53)	**<0.001**		1.06 (0.80–1.41)	0.699	
≥85	2.90 (1.87–4.49)	**<0.001**		1.19 (0.75–1.87)	0.466	
Shanghai resident (ref.: Yes)			**<0.001**			**<0.001**
No	1.61 (1.44–1.81)	**<0.001**		1.44 (1.26–1.66)	**<0.001**	
Unspecified	1.22 (1.00–1.48)	0.051		1.20 (0.94–1.52)	0.136	
Tumor location^b^ (ref.: C16: Stomach)			**<0.001**			**<0.001**
C18: Colon	0.70 (0.55–0.88)	**0.002**		0.86 (0.67–1.10)	0.225	
C20: Rectum	0.44 (0.32–0.60)	**<0.001**		1.21 (0.82–1.79)	0.328	
C25: Pancreas	6.85 (3.72–12.60)	**<0.001**		7.47 (4.07–13.70)	**<0.001**	
C34: Bronchus and lung	0.15 (0.12–0.19)	**<0.001**		20.87 (14.17–30.75)	**<0.001**	
C50: Breast	0.16 (0.11–0.23)	**<0.001**		0.02 (0.01–0.04)	**<0.001**	
Others	0.23 (0.19–0.28)	**<0.001**		0.54 (0.44–0.66)	**<0.001**	
Tumor histology^b^ (ref.: 814–838: Adenocarcinomas)			**<0.001**			**<0.001**
850–854: Ductal and lobular cancers	1.40 (1.02–1.93)	**0.035**		0.73 (0.48–1.11)	0.136	
805–808: Squamous cell cancers	0.44 (0.37–0.52)	**<0.001**		0.36 (0.29–0.44)	**<0.001**	
844–849: Cystic, mucinous, and serous cancers	1.17 (0.93–1.47)	0.195		1.20 (0.93–1.55)	0.158	
Others	0.92 (0.73–1.17)	0.512		0.68 (0.52–0.89)	**0.005**	
Unspecified	0.56 (0.45–0.70)	**<0.001**		0.40 (0.30–0.52)	**<0.001**	
History of hypertension (ref.: No)						
Yes	1.18 (0.92–1.52)	0.189		1.07 (0.79–1.45)	0.660	
History of diabetes (ref.: No)						
Yes	1.21 (0.86–1.71)	0.285		0.84 (0.56–1.26)	0.399	
History of cataract (ref.: No)						
Yes	1.07 (0.87–1.31)	0.533		1.28 (1.01–1.64)	**0.045**	
History of coronary heart disease (ref.: No)						
Yes	0.92 (0.72–1.18)	0.507		1.13 (0.84–1.53)	0.416	
History of arrhythmia (ref.: No)						
Yes	1.19 (0.90–1.59)	0.230		1.11 (0.79–1.57)	0.543	
History of stroke (ref.: No)						
Yes	0.86 (0.63–1.17)	0.334		1.09 (0.76–1.58)	0.638	
History of cirrhosis (ref.: No)						
Yes	3.44 (2.05–5.75)	**<0.001**		0.70 (0.40–1.20)	0.194	
History of major abdominal surgery (ref.: No)						
Yes	0.88 (0.76–1.03)	0.118		1.03 (0.85–1.24)	0.785	
History of intake of antihypertensive drug (ref.: No)						
Yes	0.93 (0.72–1.19)	0.551		0.88 (0.65–1.19)	0.391	
History of intake of hypoglycemic drug (ref.: No)						
Yes	1.03 (0.73–1.44)	0.874		1.28 (0.86–1.91)	0.223	
History of intake of anticoagulant (ref.: No)						
Yes	1.88 (1.42–2.48)	**<0.001**		1.09 (0.78–1.53)	0.609	
History of intake of analgesic (ref.: No)						
Yes	1.28 (0.61–2.71)	0.517		0.96 (0.42–2.21)	0.925	
History of intake of sedative (ref.: No)						
Yes	0.65 (0.40–1.03)	0.067		1.27 (0.71–2.26)	0.416	
History of intake of psychotropic drug (ref.: No)						
Yes	1.54 (0.92–2.57)	0.098		0.82 (0.44–1.51)	0.514	
Body mass index on first admission (as continuous)	0.99 (0.97–1.01)	0.161		0.97 (0.95–0.99)	**0.003**	
Weight loss on first admission (ref.: No)						
Yes	1.70 (1.43–2.03)	**<0.001**		0.93 (0.76–1.13)	0.440	
Percentage of reduced food intake within 1 week of first admission (%; ref.: 0)			**<0.001**			0.524
1–50	1.93 (1.43–2.60)	**<0.001**		1.24 (0.92–1.68)	0.166	
51–75	2.77 (1.37–5.60)	**0.005**		1.10 (0.61–2.01)	0.750	
76–100	4.00 (0.90–17.80)	0.069		1.49 (0.42–5.36)	0.538	
Basic diet on first admission (ref.: Common diet)			0.671			**<0.001**
Soft or semi‐fluid diet	1.05 (0.86–1.30)	0.629		1.21 (0.97–1.51)	0.097	
Others	1.10 (0.87–1.38)	0.425		1.78 (1.35–2.36)	**<0.001**	
Low‐salt diet on first admission (ref.: No)						
Yes	0.54 (0.43–0.69)	**<0.001**		0.85 (0.64–1.14)	0.276	
Diabetes diet on first admission (ref.: No)						
Yes	0.69 (0.52–0.92)	**0.010**		0.83 (0.60–1.16)	0.273	
Risk of malnutrition on first admission (ref.: No)						
Yes	1.02 (0.86–1.23)	0.799		0.89 (0.72–1.10)	0.290	
Risk of falling on first admission (ref.: No)						
Yes	1.05 (0.86–1.29)	0.644		1.28 (0.99–1.66)	0.058	
Barthel index for self‐care ability on first admission (as continuous)	0.99 (0.98–1.00)	**0.035**		1.01 (1.00–1.02)	0.143	

Abbreviations: −, not estimable; CI, confidence interval; OR, odds ratio; ref., reference.

^a^ The ORs and the corresponding 95% CI for the associations of ≥ versus <10 days of first hospital stay and ≥ versus <33,000 RMB as costs for first hospitalization with the factors listed in the first column of the table were estimated using the multivariable‐adjusted logistic regression model with mutual adjustment for the variables listed in the first column. Admission and resection referred to those to/in Ruijin Hospital, Shanghai Jiao Tong University School of Medicine in Shanghai, China. *p* values which are <0.05 are shown in bold and indicate statistical significance.

^b^ Coded according to the International Classification of Diseases for Oncology, Third Edition (ICD‐O‐3).

## 4 DISCUSSION

In this institution‐based study, we reported the overall and gender‐ and resection‐stratified characteristics and in‐hospital outcomes of hospitalized older patients with cancer, using the 5‐year individual‐level data of about 21,000 patients with invasive solid cancers who were ≥65 years from a top‐ranked tertiary hospital in Shanghai, China. We focused on the duration, cost, and death during first hospitalization and explored the associated factors, suggesting potentially modifiable places in clinical practice for better geriatric care.

The number of patients with cancer ≥65 years in 2020 was approximately 1.4 times that in 2016. Notably, about 12% of the older patients were admitted through the emergency pathway and had more often longer first hospital stay with higher costs and greater possibility of in‐hospital mortality; resected patients had more often emergency admission. In older patients, some cancers may only be detected after emergency complications occur. Emergency surgery may not provide the optimal benefits to patients with cancer. 45% of the older patients were resected in our hospital. While resection could benefit selected older patients with cancer, careful peri‐treatment geriatric assessments are vital.^10^ Half of the resected patients underwent surgery using the minimally invasive or robotic approach, which may especially benefit older patients who are often frailer.^27^

About half and 1/5 of the older patients had hypertension and diabetes. Patients with several comorbidities, including diabetes and cirrhosis, had more frequently longer first hospitalization with higher costs. Older patients with cancer more frequently have multiple severer and more complicated comorbidities, which require meticulous care to ensure better in‐hospital and long‐term outcomes.^29^, ^30^, ^31^ Around 1/5 and 1/10 of the older patients had weight loss and reduced food intake, respectively, both of which were associated with more often longer hospitalization and higher costs. Patients with reduced food intake also died more frequently during first hospitalization and may warrant special attention. No publications on the associations between reduced food intake and in‐hospital outcomes specifically in older patients with cancer have been identified. The multicenter NOURISH Point Prevalence Study^32^ showed that in patients undergoing resection for upper gastrointestinal cancer with a mean age of 67 years, reduced food intake was independently associated with preoperative malnutrition and unintentional weight loss, both of which were independently associated with length of stay but not with complications. Patients with gastrointestinal or lung cancer reporting a reduced food intake had more symptoms.^33^ Reduced food intake was significantly associated with distress in patients with advanced head‐and‐neck cancer, with a mean age of 58 years.^34^ These highlight the importance of early, meticulous, and individualized nutritional screening, assessment, and support for cancer patients, especially those with reduced food intake,^35^ and our findings also indicate reduced food intake as a new important risk factor, potentially alerting in‐hospital mortality in older cancer patients. Notably, about 30% of the patients had risks of malnutrition on first admission. For older patients, careful nutrition evaluations and supports are strongly warranted.

In our hospital, the median duration and cost of first hospitalization were 9 days and 32,000 RMB, respectively, and the positive correlation between duration and cost was especially prominent in patients ≥85 years and for pancreas cancers. Notably, there was still a proportion of costs that could not be covered by insurance, and further efforts are needed to reduce this part. Duration decreased in more recent years, suggesting improvements in in‐hospital geriatric care in our hospital, which might also partly increase the cost of hospitalization. The increased cost of first hospitalization could also be due to the enrichment in the kinds of treatment modalities and the more frequent use of certain treatment modalities (e.g., immunotherapy and targeted therapy). Notably, the median cost not covered by insurance for first hospitalization decreased markedly from 22,448 RMB in 2016 to 7105 RMB in 2020, and the ratio of the cost not covered by insurance for first hospitalization to the total cost of first hospitalization decreased dramatically from 98% to 21%. Further studies are needed to explore other underlying reasons. The cost of hospitalization for a certain cancer could be influenced by various factors not adjusted for, including chemotherapy, immunotherapy, room, and nursing staff.

Notably, patients with histories of anticoagulant intake had more frequently longer first hospitalization, which is contrary to the situation for sedative intake. Histories of analgesic and sedative intakes were both associated with less frequently higher costs. Patients with histories of psychotropic drug intake had similar length of stay, lower hospitalization costs which might be primarily driven by surgical interventions, but markedly elevated risks of in‐hospital death. It might be partly because these patients had more often dementia or other psychiatric comorbidities (data on such comorbidities were not readily available) as reflected by the corresponding medication history and significantly less often received surgical interventions for either curative or palliative intent compared to those who were not on any psychotropic medication (36% vs. 45%). Further studies are needed to address the underlying reasons for the differences. Patients on psychotropic medications may be a population where geriatric oncology may play a meaningful role. Drug–drug interactions and the possible incompatibility between anticancer and other medications also need to be carefully considered. Some medication use may not be appropriate.^36^, ^37^, ^38^ It would be interesting to further validate the impacts of use of certain medications on in‐hospital outcomes, and the association between sedative intake and in‐hospital outcomes may suggest the importance of psychological care for older patients.

0.6% and 1.1% of patients died during first and any hospitalization, respectively. In‐hospital deaths occurred most frequently in those ≥85 years. Perioperative deaths occurred scarcely (0.2%) for older patients with cancer in our hospital; for resection, older patients usually underwent strict selection processes and were generally fitter than unresected patients.^8^ Of note, history of hypertension was associated with markedly increased risks of in‐hospital mortality, while intake of antihypertensive drugs appeared to be obviously protective. This highlights the importance of peri‐treatment blood pressure control for older patients to ensure good in‐hospital outcomes. Patients with worse self‐care ability died more frequently during first hospitalization, which could be due to the greater vulnerability and frailty. Factors associated with in‐hospital events as revealed by multivariable analysis could help identify the subgroup of older patients requiring more meticulous management to improve in‐hospital outcomes.

About half of all patients resided in Shanghai regardless of registered permanent residence in Shanghai and medical insurance status, as our hospital is located in Shanghai. Patients from outside of Shanghai, which comprised a larger proportion in males than in females, died less often during hospitalization, possibly due to that they might be medically fitter for longer distance travel to Shanghai than local residents. However, patients from outside of Shanghai stayed longer and accordingly paid more for first hospitalization, and the possible reasons might include the following: First, regardless of performance status, the disease conditions of patients from outside of Shanghai might be more complex with initial diagnosis and/or management in their local hospital; second, since it would be less convenient for people from outside of Shanghai to come to our hospital, which possibly had better medical resources, they might seek for more comprehensive and meticulous medical care during hospitalization, and physicians also tried to offer more medical help at a time causing longer hospital stay, potentially to reduce the number of travels to Shanghai of the patients; third, it might be related to medical insurance, and for some patients from outside of Shanghai, it might be easier for them to have reimbursement for medical costs during hospitalization.

Comprehensive geriatric evaluations and geriatric screening methods may assist with identifying patients who are at the highest risk of poor outcomes and with better allocating anticancer treatment for them. The application of evidence‐based strategies to optimize geriatric management can improve satisfactions of patients, physicians, and caregivers and enhance outcomes in the geriatric population.^3^, ^6^

A retrospective cohort study^12^ evaluating the in‐hospital mortality and length of stay in older patients with solid malignancies hospitalized between 1995 and 2003 using the University Health System Consortium database revealed an overall mortality rate of 7%, which is higher than the mortality rate during first or any hospitalization in our study (1%); the study further found that male gender was significantly associated with an increased risk of mortality, while we did not detect a significant difference in mortality between genders. A retrospective study^13^ assessing the inpatient mortality in elderly patients with metastatic brain cancers undergoing resection during 1998–2005 using the US Nationwide Inpatient Sample (NIS) database showed that the inpatient mortality was 4%, that the mean length of stay was 9 days, and that the mean total charges were $57,596. Another retrospective study^14^ investigating the in‐hospital mortality in geriatric patients with colorectal cancer undergoing resection between 2005 and 2014 using the US NIS database showed that emergent admission was most prominently associated with an increased risk of mortality (OR = 3.01), followed by age ≥85 years (OR = 2.58). Similarly, our pan‐solid cancer analysis also showed that compared to outpatient admission, emergency admission was significantly associated with an increased risk of death during first hospitalization (OR = 2.46); patients ≥85 years had a significantly higher risk of mortality than those aged 65–69 years (OR = 2.28). A retrospective study^15^ also using the NIS database explored the 30‐day in‐hospital mortality in older patients with pancreatic ductal adenocarcinoma undergoing pancreatoduodenectomy between 2007 and 2010 and found that the overall in‐hospital mortality rate was 3%; compared to <76 years, age ≥76 years was significantly associated with higher risks of in‐hospital mortality (4% vs. 3%; OR = 1.46) and longer in‐hospital stay (OR = 1.09). We also found that compared to patients aged 65–69 years, older patients had significantly longer first hospital stay, with ORs of ≥ versus <10 days for 70–74, 75–79, 80–84, and ≥85 years being 1.17, 1.35, 1.58, and 1.60, respectively. A study^16^ assessed the cost of operative hospitalizations in elderly patients with cancer using the 2014 NIS data did not reveal a significant association between age and hospitalization cost, but we found that older patients did have a higher cost (e.g., compared to patients aged 65–69 years, the ORs of ≥ vs. <33,000 RMB for 75–79 and ≥85 years were 1.23 and 1.38, respectively).

The PSM method had an only minor influence on the inter‐gender differences in a few in‐hospital outcomes, which were not the focus of this study and did not influence the comparisons according to resectional surgery status. Thus, the conclusions were not changed by the PSM analyses.

Our observational study has several limitations. While data were prospectively collected in electronic medical records, analyses were carried out retrospectively. We analyzed data stratified by surgery; nonsurgical therapies were not reported due to low sensitivity and large proportion of missing values. Information on room and nursing staff was not available. We did not report TNM stages, considering that they are defined differently and not comparable across different cancers. The proportion of unspecified tumor histology was especially high for unresected patients. Only in‐hospital outcomes were collected, which are the focus of our study, and events out of hospital and in the longer term need to be investigated in further studies. Our findings based on data from a China tertiary hospital may not be generalizable to institutions of other levels or in other countries. Data on costs spanned 5 years (2016–2020), and there was no price adjustment policy during this period. Statistical significance does not necessarily indicate clinical significance. While there was a statistical significance for some comparisons (e.g., history of arrhythmia by gender) in this study with a large case number, the clinical significance or relevance might be limited or lacking, and the *P* values for such comparisons should be deemphasized.

While it was hypothesized that low‐salt and diabetes diets, which could help control conditions of cardiovascular diseases and diabetes, might contribute to shortening the length of first hospital stay, the association results in this observational study do not mean causality, and should be further validated in prospective and/or randomized studies. In multivariable‐adjusted modeling analyses, tumor histology and location were mutually adjusted for. The majority (79.0%) of male patients with ductal/lobular cancers had pancreatic cancer, and male breast cancer patients comprised only 1.5%. There were only 10 male breast cancer patients with ductal/lobular morphology included.

Information on distant metastasis was not readily available. This study was a pan‐solid cancer study. TNM stage was coded differently for different cancers. Stage IV cancers might include non‐metastatic cancers for some cancer types (e.g., cervical carcinoma, liver cancer, and thyroid cancer), and in gastric cancer, distant metastasis included distant lymph node involvement, positive peritoneal cytology, etc. Distant metastasis was not listed as a readily extractable uniform variable in the electronic medical records of our hospital. We did analyses stratified by resectional surgery, and resected cancers might be mostly non‐metastatic. Notably, some metastatic cancers (e.g., colorectal cancer) might still be resectable, and some non‐metastatic cancers (e.g., pancreatic cancer) might be already unresectable. Information on curative versus palliative resections was also not readily available. Resections with curative intent as described in the electronic medical records might sometimes be non‐curative (R1/2 resections). Moreover, resection margin might be defined differently across different cancers.^10^, ^39^ Information on the number of patients diagnosed with cancer during the first admission as opposed to coming in with the diagnosis already established was also not readily available. Our hospital is a tertiary referral hospital. Generally speaking, most of the patients residing outside Shanghai and/or undergoing resectional surgery came to our hospital for cancer treatment with the diagnosis already established, and most of the patients admitted through the emergency pathway were diagnosed with cancer during the first admission.

Nevertheless, our study appears to be the largest institution‐based report on features and in‐hospital outcomes of older patients with cancer in China. We comprehensively characterized patients with cancer ≥65 years from various aspects, highlighting the comorbidities, medical and medication histories, nutrition, risk assessments, and self‐care ability, and their impacts on in‐hospital outcomes, which are often not thoroughly addressed in previous studies on older patients with cancer, and performed careful stratification analyses by gender and resection. We carefully explored various factors associated with duration, cost, and mortality during first hospitalization, and the findings provide important hints suggesting potentially modifiable places in clinical practice and valuable references for further enhancing geriatric oncology care.

In conclusion, this study first described the characteristics and in‐hospital outcomes (duration, cost, and mortality) of elderly patients with solid cancer admitted to our hospital in 2016–2020, overall and stratified by gender and resectional surgery, and then further identified certain baseline patient and tumor characteristics, medical and medication histories, changes of weight and food intake, diet, and self‐care ability, which were independently associated with in‐hospital outcomes among older patients with cancer, and which should be paid special attention to. While the factors might not be easily modifiable, our study can help identify older cancer patients at higher risks of inferior in‐hospital outcomes.

## AUTHOR CONTRIBUTIONS

Conception or design: Huang L, Hu W, and Zhang J. Acquisition, analysis, or interpretation of data: Huang L, Shi Y, Wang L, Rong L, Ren Y, Xu C, Wu J, Zhang M, Zhu L, Zhang Jj, Xu X, Hu W, and Zhang J. Drafting of the manuscript: Huang L. Critical revision of the manuscript for important intellectual content: Huang L, Shi Y, Wang L, Rong L, Ren Y, Xu C, Wu J, Zhang M, Zhu L, Zhang Jj, Xu X, Hu W, and Zhang J. Statistical analysis: Huang L. Administrative, technical, or material support: Hu W and Zhang J. All authors have approved the current version of the manuscript for submission and publication.

## FUNDING INFORMATION

Our study was supported by Shanghai Pujiang Program (21PJ1409700), the Fund for Medical Center on Aging (GB2021), and the Start‐up Fund for the Introduction of High Level Talents by Ruijin Hospital, Shanghai Jiao Tong University School of Medicine. The funders had no role in study design; in the collection, analysis, or interpretation of data; in the writing of the report; or in the decision to submit the paper for publication.

## CONFLICT OF INTEREST

None exist.

## Supporting information


Appendix S1

## Data Availability

Restrictions apply to the availability of the data for this study, which were used under license, and so are not publicly available.
