# Supplementary material for: Characteristics and in‐hospital outcomes of elderly patients with cancer in a top‐ranked hospital in China, 2016–2020: Real‐world study
Source: Cancer Med. 2022 Sep 26;12(3):2885–905. doi: 10.1002/cam4.5203 (PMC9939123; doi:10.1002/cam4.5203)
Supplement: Supplementary file 1 — Appendix S1 [file CAM4-12-2885-s001.docx]

**Supplementary Materials**

**Supplementary Methods**

***Definitions of variables***

**Supplementary Results**

***Medical and medication histories***

***Nutrition, risk assessments, and self-care ability***

***Characteristics and in-hospital outcomes of resected patients on admission for surgery***

***Duration and cost of first hospitalization against age group, year of diagnosis, cancer type, and histology, with stratification by gender and resection***

**Table S1**. Characteristics of patients undergoing resectional surgery on admission for surgery

**Table S2**. Characteristics of patients who died during first or any hospitalization

**Table S3**. Patient and tumor characteristics after propensity score matching (PSM)

**Table S4**. Hospitalization, costs, and in-hospital deaths after propensity score matching (PSM)

**Supplementary Methods**

***Definitions of variables***

Shanghai resident: People residing in Shanghai regardless of registered permanent residence in Shanghai and medical insurance status.

History of major abdominal surgery: Interventional surgical procedures in the abdomen with therapeutic intent (e.g., resection) prior to the first hospitalization of each patient in our hospital.

Medications: Analgesics included opioids for therapeutic (pain-relieving) intent. Psychotropic drugs were drugs for management of psychiatric disorders.

Weight loss on first admission: weight loss prior to the first hospitalization which was determined by medical history only.

Risk of fall: Assessed using the Morse Fall Scale^1-3^.

**References**

1. Adly NN, Abd-El-Gawad WM, Abou-Hashem RM. Relationship between malnutrition and different fall risk assessment tools in a geriatric in-patient unit. Aging Clin Exp Res 2020;32:1279-87.

2. Chu JJ, Chen XJ, Shen SS, et al. A poor performance in comprehensive geriatric assessment is associated with increased fall risk in elders with hypertension: a cross-sectional study. J Geriatr Cardiol 2015;12:113-8.

3. Mamun K, Lim JK. Association between falls and high-risk medication use in hospitalized Asian elderly patients. Geriatr Gerontol Int 2009;9:276-81.

**Supplementary Results**

***Medical and medication histories***

Among all patients, the most common medical history was hypertension (49%), followed by major abdominal surgery (28%) and diabetes (19%). Accordingly, the most commonly taken drug was antihypertensive drug (45%), followed by hypoglycemic drug (16%). While there were some statistically significant differences between males and females and between patients not undergoing and undergoing resection in our hospital, the real differences in proportions were mostly small, except for that patients resected in our hospital had markedly less often histories of major abdominal surgery (21% vs 34%).

***Nutrition, risk assessments, and self-care ability***

The median body mass index (BMI) of all the patients on first admission to our hospital was 23 kg/m^2^, and 22% had weight loss, with median loss value of 4 kg and median loss duration of 2 months. 12% of patients had reduced food intake within 1 week of first admission, and 31% did not have common diet on first admission. The proportions of patients having low-salt and diabetes diets on first admission were 12% and 11%, respectively. 28% of patients had risks of malnutrition and 8% had risks of falling on first admission. The median Barthel Index score was 100.

Compared to male patients, females had less often weight loss on first admission (18% vs 24%). While there were some other statistically significant intersexual differences, they were mostly small.

Relative to patients not undergoing resection in our hospital, those resected in our hospital had less frequently weight loss on first admission (19% vs 24%), less often reduced food intake within 1 week of first admission (10% vs 13%); they had more often common diet (74% vs 64%), less often low-salt diet (10% vs 14%), and less frequently risks of malnutrition (21% vs 33%) on first admission.

***Characteristics and in-hospital outcomes of resected patients on admission for surgery***

98% of the patients undergoing resectional surgery in our hospital had only 1 resection, and 92% of the patients were resected during first hospitalization (**Table S1**). 50% of the patients underwent open resection, 47% were resected through minimally invasive approaches, and the other 3% had robotic resection. Characteristics and in-hospital outcomes of resected patients on admission for surgery were mostly very similar to those on first admission. The median surgery-associated cost was 7000 RMB, and took up on average 16% of the cost of hospitalization for surgery. Only 16 (0.2%) perioperative deaths occurred.

***Duration and cost of first hospitalization against age group, year of diagnosis, cancer type, and histology, with stratification by gender and resection***

In both genders and regardless of resection in our hospital, duration of first hospital stay increased with older ages. Cost of first hospitalization was highest in patients aged 75-79 years especially for females, and it increased with older ages especially for unresected patients. Except others cancer types, in both genders patients with pancreas cancers had the longest first hospital stay, while those with bronchus/lung cancers had the shortest stay; for unresected patients, duration of first hospital stay was longest in those with pancreas or rectum cancers, and shortest in those with stomach or bronchus/lung cancers; for resected patients, duration was longest for patients with pancreas cancers and shortest for those with breast cancers. With others cancer types excluded, cost of first hospitalization was also highest for patients with pancreas cancers, and lowest for those with breast cancers, in patients of both genders and in resected patients; for unresected patients, median cost was highest for rectum cancers and lowest for stomach cancers. After excluding others and unspecified tumor histology, in males duration of first hospitalization was longest for ductal/lobular cancers, and shortest for squamous cell cancers, and in females duration was longest for cystic/mucinous/serous cancers, and also shortest for squamous cell cancers; in both resected and unresected patients, duration was longest for ductal/lobular cancers followed by cystic/mucinous/serous cancers, and shortest for squamous cell cancers. With others and unspecified histology excluded, cost of first hospitalization was highest for ductal/lobular cancers in males and for cystic/mucinous/serous cancers in females, and was lowest for squamous cell cancers in both genders; cost was highest for cystic/mucinous/serous cancers in unresected patients and for ductal/lobular cancers in resected patients, and was lowest for squamous cell cancers regardless of resection in our hospital.

**Table S1**. Characteristics and in-hospital outcomes of patients undergoing resectional surgery on admission for surgery

| **Variable** | **Value** |
| --- | --- |
| n | 9241 |
| Times of resectional surgery, 1 | 9056 (98) |
| Resection during first hospitalization, yes | 8487 (92) |
| Resection approach |  |
| Open | 4608 (50) |
| Minimally invasive | 4348 (47) |
| Robotic | 285 (3) |
| Weight on admission for surgery (kg) | 63±11; 63 (56-70) |
| Body mass index on admission for surgery (kg/m^2^) | 23±3; 23 (21-25) |
| Weight loss on admission for surgery, yes | 1705 (18) |
| Weight loss value on admission for surgery (kg) | 4±3; 4 (2-5) |
| Weight loss percentage on admission for surgery (%) | 4±3; 4 (2-5) |
| Weight loss duration on admission for surgery (months) | 2±1; 2 (1-3) |
| Percentage of reduced food intake within 1 week of admission for surgery (%) |  |
| 0 | 8361 (91) |
| 1-50 | 627 (7) |
| 51-75 | 179 (2) |
| 76-100 | 60 (1) |
| Basic diet on admission for surgery |  |
| Common diet | 6874 (74) |
| Soft or semi-fluid diet | 1048 (11) |
| Others | 1319 (14) |
| Low-salt diet on admission for surgery, yes | 797 (9) |
| Diabetes diet on admission for surgery, yes | 782 (8) |
| Risk of malnutrition on admission for surgery, yes | 1995 (22) |
| Risk of falling on admission for surgery, yes | 737 (8) |
| Barthel index for self-care ability on admission for surgery | 98±9; 100 (100-100) |
| Days of hospital stay for surgery | 15±10; 13 (8-18) |
| Costs of hospitalization for surgery (×1000 RMB) | 53±34; 46 (32-65) |
| Surgery-associated costs (×1000 RMB) | 10±9; 7 (6-9) |
| Surgery-associated costs/costs of hospitalization for surgery (%) | 20±11; 16 (13-24) |
| Surgery-associated costs/total costs of any hospitalization (%) | 18±12; 15 (10-22) |
| Costs not covered by insurance for hospitalization for surgery (×1000 RMB) | 31±31; 24 (9-45) |
| Costs not covered by insurance for hospitalization for surgery/costs of hospitalization for surgery (%) | 58±40; 50 (19-100) |
| Perioperative death, yes | 16 (<1) |

**Table S2**. Characteristics of patients who died during first or any hospitalization

| **Variable** | **Death during first hospitalization** | **Death during any hospitalization** |
| --- | --- | --- |
| n | 118 | 228 |
| ***Baseline patient and tumor characteristics*** |  |  |
| Year of diagnosis |  |  |
| 2016 | 32 (27) | 76 (33) |
| 2017 | 25 (21) | 51 (22) |
| 2018 | 21 (18) | 42 (18) |
| 2019 | 19 (16) | 32 (14) |
| 2020 | 21 (18) | 27 (12) |
| Gender, male | 65 (55) | 131 (57) |
| Age (years) |  |  |
| As continuous | 76±7; 76 (69-82) | 74±7; 73 (68-79) |
| 65-69 | 32 (27) | 78 (34) |
| 70-74 | 20 (17) | 50 (22) |
| 75-79 | 27 (23) | 45 (20) |
| 80-84 | 20 (17) | 28 (12) |
| ≥85 | 19 (16) | 27 (12) |
| Residence in Shanghai |  |  |
| Yes | 82 (69) | 163 (71) |
| No | 11 (9) | 15 (6) |
| Unspecified | 25 (21) | 50 (22) |
| Admission, emergency | 40 (34) | 51 (22) |
| Tumor location |  |  |
| C16: Stomach | 16 (14) | 32 (14) |
| C18: Colon | 11 (9) | 23 (10) |
| C20: Rectum | 1 (1) | 8 (4) |
| C25: Pancreas | 24 (20) | 36 (16) |
| C34: Bronchus and lung | 23 (19) | 51 (22) |
| C50: Breast | 1 (1) | 4 (2) |
| Others | 42 (36) | 74 (32) |
| Tumor histology |  |  |
| 814-838: Adenocarcinomas | 49 (42) | 110 (48) |
| 850-854: Ductal and lobular cancers | 10 (8) | 21 (9) |
| 805-808: Squamous cell cancers | 8 (7) | 15 (7) |
| 844-849: Cystic, mucinous, and serous cancers | 2 (2) | 7 (3) |
| Others | 14 (12) | 23 (10) |
| Unspecified | 35 (30) | 52 (23) |
| Resectional surgery, yes | 16 (14) | 36 (16) |
| ***Medical and medication histories*** |  |  |
| History of hypertension, yes | 75 (64) | 128 (56) |
| History of diabetes, yes | 34 (29) | 52 (23) |
| History of cataract, yes | 21 (18) | 31 (14) |
| History of coronary heart disease, yes | 9 (8) | 18 (8) |
| History of arrhythmia, yes | 11 (9) | 22 (10) |
| History of stroke, yes | 12 (10) | 22 (10) |
| History of cirrhosis, yes | 11 (1) | 1 (<1) |
| History of major abdominal surgery, yes | 40 (34) | 87 (38) |
| History of intake of antihypertensive drug, yes | 64 (54) | 116 (51) |
| History of intake of hypoglycemic drug, yes | 26 (22) | 42 (18) |
| History of intake of anticoagulant, yes | 5 (4) | 12 (5) |
| History of intake of analgesic, yes | 12 (10) | 26 (11) |
| History of intake of sedative, yes | 0 (0) | 7 (3) |
| History of intake of psychotropic drug, yes | 12 (10) | 16 (7) |
| ***Changes of weight and food intake, diet, risk assessments, and self-care ability*** | | |
| Height (cm) | 165±8; 165 (158-172) | 165±8; 165 (158-172) |
| Weight on first admission (kg) | 59±13; 56 (50-67) | 59±12; 57 (50-67) |
| Body mass index on first admission (kg/m^2^) | 21±4; 21 (18-24) | 22±4; 22 (19-24) |
| Weight loss on first admission, yes | 44 (37) | 80 (35) |
| Weight loss value on first admission (kg) | 5±3; 5 (4-6) | 5±3; 5 (3-6) |
| Weight loss percentage on first admission (%) | 6±3; 5 (4-6) | 5±3; 5 (3-6) |
| Weight loss duration on first admission (months) | 2±1; 2 (1-3) | 2±1; 2 (1-3) |
| Percentage of reduced food intake within 1 week of first admission (%) |  |  |
| 0 | 69 (58) | 155 (68) |
| 1-50 | 20 (17) | 33 (14) |
| 51-75 | 22 (19) | 30 (13) |
| 76-100 | 7 (6) | 10 (4) |
| Basic diet on first admission |  |  |
| Common diet | 55 (47) | 123 (54) |
| Soft or semi-fluid diet | 19 (16) | 45 (20) |
| Others | 44 (37) | 60 (26) |
| Low-salt diet on first admission, yes | 16 (14) | 31 (14) |
| Diabetes diet on first admission, yes | 16 (14) | 27 (12) |
| Risk of malnutrition on first admission, yes | 67 (57) | 125 (55) |
| Risk of falling on first admission, yes | 43 (36) | 67 (29) |
| Barthel index for self-care ability on first admission | 70±33; 80 (50-100) | 78±30; 100 (60-100) |
| ***Hospitalization, costs, and in-hospital deaths*** |  |  |
| Times of hospitalization | - | 5±8; 1 (1-4) |
| Days of first hospital stay |  |  |
| As continuous | 33±39; 21 (10-40) | 24±36; 13 (6-28) |
| ≥10 | 91 (77) | 142 (62) |
| Days of total hospital stay | 33±39; 21 (10-40) | 50±62; 31 (14-67) |
| Costs of first hospitalization (×1000 RMB) |  |  |
| As continuous | 101±128; 56 (22-127) | 69±105; 30 (13-77) |
| ≥33 | 72 (61) | 107 (47) |
| Total costs of any hospitalization (×1000 RMB) | 101±128; 56 (22-127) | 132±152; 77 (31-175) |
| Costs not covered by insurance for first hospitalization (×1000 RMB) | 40±72; 15 (1-49) | 29±55; 10 (1-33) |
| Costs not covered by insurance for first hospitalization/costs of first hospitalization (%) | 49±45; 23 (3-97) | 53±44; 57 (5-97) |
| Total costs not covered by insurance for any hospitalization (×1000 RMB) | 40±72; 15 (1-49) | 53±93; 20 (5-70) |
| Total costs not covered by insurance for any hospitalization/total costs of any hospitalization (%) | 49±45; 23 (3-97) | 47±42; 26 (5-95) |
| Death during first hospitalization, yes | 118 (100) | 118 (52) |

**Table S3**. Patient and tumor characteristics after propensity score matching (PSM)^1^

| **Variables** | **Gender** | | | **Resectional surgery** | | |
| --- | --- | --- | --- | --- | --- | --- |
|  | Male | Female | *P* | No | Yes | *P* |
| ***Baseline patient and tumor characteristics*** |  |  |  |  |  |  |
| n | 2544 | 2544 |  | 5320 | 5320 |  |
| Year of diagnosis |  |  |  |  |  |  |
| 2016 | 479 (19) | 467 (18) | 0.936 | 884 (17) | 838 (16) | 0.684 |
| 2017 | 432 (17) | 440 (17) |  | 953 (18) | 984 (19) |  |
| 2018 | 476 (19) | 475 (19) |  | 991 (19) | 968 (18) |  |
| 2019 | 546 (22) | 530 (21) |  | 1141 (21) | 1160 (22) |  |
| 2020 | 611 (24) | 632 (25) |  | 1351 (25) | 1370 (26) |  |
| Gender, male | 2544 (100) | 0 (0) | - | 3187 (60) | 3162 (59) | 0.635 |
| Age (years) |  |  |  |  |  |  |
| As continuous | 71±6 | 71±6 | 0.614 | 72±6 | 71±6 | 0.193 |
| 65-69 | 1226 (48) | 1244 (49) | 0.884 | 2436 (46) | 2487 (47) | 0.725 |
| 70-74 | 705 (28) | 707 (28) |  | 1502 (28) | 1503 (28) |  |
| 75-79 | 360 (14) | 341 (13) |  | 808 (15) | 785 (15) |  |
| 80-84 | 184 (7) | 176 (7) |  | 396 (7) | 386 (7) |  |
| ≥85 | 69 (3) | 76 (3) |  | 178 (3) | 159 (3) |  |
| Residence in Shanghai |  |  |  |  |  |  |
| Yes | 1145 (45) | 1170 (46) | 0.771 | 2545 (48) | 2532 (48) | 0.932 |
| No | 1174 (46) | 1150 (45) |  | 2306 (43) | 2309 (43) |  |
| Unspecified | 225 (9) | 224 (9) |  | 469 (9) | 479 (9) |  |
| Resectional surgery, yes | 1232 (48) | 1175 (46) | 0.116 | 0 (0) | 5320 (100) | - |
| ***Medical and medication histories*** |  |  |  |  |  |  |
| History of hypertension, yes | 1228 (48) | 1227 (48) | 1.000 | 2655 (50) | 2674 (50) | 0.727 |
| History of diabetes, yes | 465 (18) | 487 (19) | 0.450 | 1024 (19) | 1029 (19) | 0.922 |
| History of cataract, yes | 214 (8) | 231 (9) | 0.427 | 484 (9) | 474 (9) | 0.761 |
| History of coronary heart disease, yes | 153 (6) | 143 (6) | 0.590 | 338 (6) | 316 (6) | 0.397 |
| History of arrhythmia, yes | 106 (4) | 106 (5) | 1.000 | 235 (4) | 238 (5) | 0.925 |
| History of stroke, yes | 108 (4) | 97 (4) | 0.476 | 231 (4) | 209 (4) | 0.307 |
| History of cirrhosis, yes | 46 (2) | 38 (1) | 0.441 | 66 (1) | 53 (1) | 0.269 |
| History of major abdominal surgery (prior to first hospitalization), yes | 716 (28) | 719 (28) | 0.950 | 1356 (26) | 1328 (25) | 0.547 |
| History of intake of antihypertensive drug, yes | 1152 (45) | 1149 (45) | 0.955 | 2491 (47) | 2520 (47) | 0.587 |
| History of intake of hypoglycemic drug, yes | 409 (16) | 420 (17) | 0.704 | 906 (17) | 906 (17) | 1.000 |
| History of intake of anticoagulant, yes | 142 (6) | 136 (5) | 0.758 | 293 (6) | 263 (5) | 0.206 |
| History of intake of analgesic, yes | 74 (3) | 63 (3) | 0.386 | 83 (2) | 72 (1) | 0.418 |
| History of intake of sedative, yes | 55 (2) | 51 (2) | 0.768 | 105 (2) | 96 (2) | 0.569 |
| History of intake of psychotropic drug, yes | 35 (1) | 36 (1) | 1.000 | 75 (1) | 69 (1) | 0.675 |
| ***Changes of weight and food intake, diet, risk assessments, and self-care ability*** | | | |  |  |  |
| Body mass index on first admission (kg/m^2^) | 23.0±3.3 | 23.0±3.5 | 0.642 | 23.1±3.4 | 23.2±3.3 | 0.038 |
| Weight loss prior to the first hospitalization^3^, yes | 580 (23) | 570 (22) | 0.763 | 1238 (23) | 1179 (22) | 0.180 |
| Percentage of reduced food intake within 1 week of first admission (%)^5^ |  |  |  |  |  |  |
| 0 | 2206 (87) | 2203 (87) | 0.744 | 4602 (87) | 4662 (88) | 0.316 |
| 1-50 | 239 (9) | 231 (9) |  | 509 (10) | 459 (9) |  |
| 51-75 | 78 (3) | 91 (4) |  | 160 (3) | 157 (3) |  |
| 76-100 | 21 (1) | 19 (1) |  | 49 (1) | 42 (1) |  |
| Basic diet on first admission |  |  |  |  |  |  |
| Common diet | 1765 (69) | 1760 (69) | 0.258 | 3694 (69) | 3778 (71) | 0.155 |
| Soft or semi-fluid diet | 388 (15) | 358 (14) |  | 712 (13) | 655 (12) |  |
| Others | 391 (15) | 426 (17) |  | 914 (17) | 887 (17) |  |
| Low-salt diet on first admission, yes | 242 (10) | 263 (10) | 0.348 | 628 (12) | 626 (12) | 0.976 |
| Diabetes diet on first admission, yes | 246 (10) | 267 (11) | 0.352 | 577 (11) | 551 (10) | 0.431 |
| Risk of malnutrition on first admission^6^, yes | 716 (28) | 706 (28) | 0.779 | 1447 (27) | 1400 (26) | 0.314 |
| Risk of falling on first admission^7^, yes | 211 (8) | 217 (9) | 0.801 | 448 (8) | 452 (9) | 0.917 |
| Barthel index for self-care ability on first admission | 96±13 | 96±13 | 0.468 | 97±10 | 97±11 | 0.066 |

^1^Categorical variables are shown as count (percentage [%]), and are compared between groups using *χ^2^* or Fisher’s exact test where appropriate. Continuous variables are shown as mean ± standard deviation; median (interquartile range), and are compared between groups using Wilcoxon rank-sum test (Mann-Whitney *U* test). Admission and resection referred to those to/in Ruijin Hospital, Shanghai Jiao Tong University School of Medicine in Shanghai, China.

^2^Coded according to the International Classification of Diseases for Oncology, Third Edition (ICD-O-3).

^3^Unknown weight loss prior to the first hospitalization: Total, 109 (1%); male, 62 (1%); female, 47 (1%); unresected, 95 (1%); resected, 14 (<1%).

^4^Calculated for cases with weight loss on first admission only.

^5^Unknown percentage of reduced food intake within 1 week of first admission: Total, 110 (1%); male, 62 (1%); female, 48 (1%); unresected, 96 (1%); resected, 14 (<1%).

^6^Unknown risk of malnutrition on first admission: Total, 109 (1%); male, 62 (1%); female, 47 (1%); unresected, 95 (1%); resected, 14 (<1%).

^7^Unknown risk of falling on admission for surgery: Total, 109 (1%); male, 62 (1%); female, 47 (1%); unresected, 95 (1%); resected, 14 (<1%).

**Table S4**. Hospitalization, costs, and in-hospital deaths after propensity score matching (PSM)^1^

| **Variable** | **Gender** | | | **Resectional surgery** | | |
| --- | --- | --- | --- | --- | --- | --- |
|  | Male | Female | *P* | No | Yes | *P* |
| n | 2544 | 2544 |  | 5320 | 5320 |  |
| Number of hospitalizations | 1 (1-2) | 1 (1-2) | 0.087 | 1 (1-3) | 1 (1-2) | <0.001 |
| Days of first hospital stay |  |  |  |  |  |  |
| As continuous | 10 (5-16) | 9 (5-15) | 0.004 | 9 (5-15) | 12 (8-18) | <0.001 |
| ≥10 | 1319 (52) | 1230 (48) | 0.014 | 2508 (47) | 3440 (65) | <0.001 |
| Days of total hospital stay | 14 (8-24) | 13 (7-22) | 0.001 | 13 (7-24) | 15 (10-24) | <0.001 |
| Costs of first hospitalization (×1000 RMB) |  |  |  |  |  |  |
| As continuous | 33 (12-57) | 32 (12-51) | 0.027 | 24 (11-48) | 45 (28-65) | <0.001 |
| ≥33 | 1265 (50) | 1214 (48) | 0.161 | 2151 (40) | 3633 (68) | <0.001 |
| Total costs of any hospitalization (×1000 RMB) | 47 (26-78) | 44 (22-75) | 0.002 | 41 (19-77) | 54 (36-84) | <0.001 |
| Costs not covered by insurance for first hospitalization (×1000 RMB) | 13 (3-35) | 12 (2-34) | 0.126 | 11 (2-27) | 21 (7-44) | <0.001 |
| Costs not covered by insurance for first hospitalization/costs of first hospitalization (%) | 65 (12-100) | 68 (10-100) | 0.811 | 52 (6-100) | 50 (16-100) | <0.001 |
| Total costs not covered by insurance for any hospitalization (×1000 RMB) | 19 (5-45) | 18 (4-44) | 0.094 | 15 (3-39) | 27 (9-52) | <0.001 |
| Total costs not covered by insurance for any hospitalization/total costs of any hospitalization (%) | 51 (13-100) | 50 (11-100) | 0.623 | 43 (7-100) | 45 (16-100) | <0.001 |
| Death during first hospitalization, yes | 13 (1) | 19 (1) | 0.375 | 43 (1) | 12 (<1) | <0.001 |
| Death during any hospitalization, yes | 28 (1) | 33 (1) | 0.606 | 94 (2) | 27 (1) | <0.001 |

^1^Categorical variables are shown as count (percentage [%]), and were compared between groups using *χ^2^* or Fisher’s exact test where appropriate. Continuous variables are shown as median (interquartile range), and were compared between groups using Wilcoxon rank-sum test (Mann-Whitney *U* test). Admission and resection referred to those to/in Ruijin Hospital, Shanghai Jiao Tong University School of Medicine in Shanghai, China.
